# Supplementary material for: Coupled multiferroic domain switching in the canted conical spin spiral system Mn2GeO4
Source: Nat Commun. 2017 Jun 5;8:15457. doi: 10.1038/ncomms15457 (PMC5465321; doi:10.1038/ncomms15457)
Supplement: Supplementary Information — Supplementary Figure, Supplementary Tables, Supplementary Note and Supplementary References [file ncomms15457-s1.pdf]

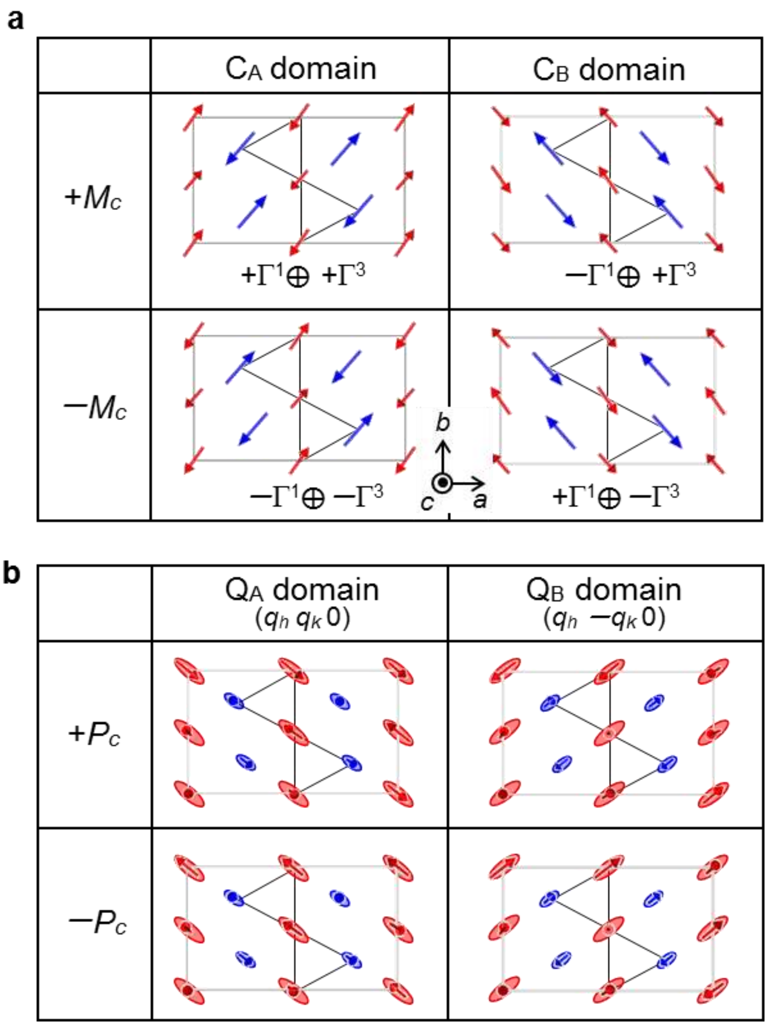

Supplementary Figure 1 | Magnetic domains in the multiferroic state of Mn_2GeO_4. (**a**) Ferromagnetic C domains ascribed to the commensurate component described by a combination of the two irreducible representations Γ^1 and Γ^3. The sign of M_c denotes the direction of spontaneous magnetization along *c* in the respective domains. Coloured arrows denote the commensurate component of Mn spins. (**b**) Ferroelectric Q domains ascribed to the incommensurate spin-spiral component with the two configurational propagation vectors (q_h q_k 0) and (q_h $-q_k$ 0). The sign of P_c denotes the direction of spontaneous polarization along *c* in the respective domains. Coloured arrows and ellipses denote the incommensurate component of Mn spins and the basal plane of the spin cone formed by Mn spins aligned along the *b* direction, respectively.

Supplementary Table 1 | Space group symmetry operations for Mn_2GeO_4.

1	(x, y, z)	$\bar{1}$	$(\bar{x}, \bar{y}, \bar{z})$
m_x	$\left(\bar{x}+\frac{1}{2}, y+\frac{1}{2}, z+\frac{1}{2}\right)$	2_x	$\left(x+\frac{1}{2}, \bar{y}+\frac{1}{2}, \bar{z}+\frac{1}{2}\right)$
m_y	$\left(x, \bar{y}+\frac{1}{2}, z\right)$	2_y	$\left(\bar{x}, y+\frac{1}{2}, \bar{z}\right)$
m_z	$\left(x+\frac{1}{2}, y, \bar{z}+\frac{1}{2}\right)$	2_z	$\left(\bar{x}+\frac{1}{2}, \bar{y}, z+\frac{1}{2}\right)$

Symmetry operations of the *Pnma* space group and their action on general positions within the unit cell. Here, the operations 1 and $\bar{1}$ denote the identity and the inversion about the origin, respectively. The operations m_x, m_y, and m_z denote the mirror (or glide) operations perpendicular to the x, y, and z axes, respectively, while 2_x, 2_y, and 2_z represent a twofold rotation (or screw) about the x, y, and z axes, respectively. The coordinates describe how an object located at (x,y,z) is transformed by the respective operations, where x, y, and z are relative (i.e. crystallographic) coordinates.

Supplementary Table 2 | Domain fractions after various field cooling conditions.

Condition before measurement	Domain fraction				M_c	P_c
	C_A	Q_A	C_B	Q_B		
ZFC	0.458(9)	0.473(2)	0.542(9)	0.527(2)	±	±
+*E*+*B* cool	0.60(1)	0.600(3)	0.40(1)	0.400(3)	+	+
−*B* sweep	0.33(1)	0.371(2)	0.67(1)	0.629(2)	−	−
+*E*−*B* cool	0.61(1)	0.617(2)	0.39(1)	0.383(2)	−	+
+*B* sweep	0.31(1)	0.349(2)	0.69(1)	0.651(2)	+	−
−*E*+*B* cool	0.49(1)	0.479(3)	0.51(1)	0.521(3)	+	−
−*B* sweep	0.42(1)	0.460(2)	0.58(1)	0.540(2)	−	+
−*E*−*B* cool	0.60(1)	0.557(4)	0.40(1)	0.443(4)	−	−
+*B* sweep	0.38(1)	0.431(3)	0.62(1)	0.569(3)	+	+
−*E* cool	0.505(9)	0.509(3)	0.495(9)	0.491(3)	±	−
+*E* cool	0.481(7)	0.483(4)	0.519(7)	0.517(4)	±	+
+*B* cool	0.67(1)	0.653(3)	0.33(1)	0.347(3)	+	±
−*B* sweep	0.361(6)	0.383(2)	0.639(6)	0.617(2)	−	±

The fractions were obtained by unpolarized neutron diffraction measurements at TriCS, PSI and refinements of the data by using the FullProf program. Here, "ZFC" denotes the zero-field-cooling condition, and "+*E*+*B* cool" means the field-cooled condition with E_{cool} = +3 MV m^{-1} and B_{cool} = +1.5 T. "+*B*-sweep" denotes that *B* was swept to +1.5 T once after the cooling procedure of the upper row and then set to zero before the measurement. All the measurements were done in the absence of *E* and *B*. The signs of M_c and P_c denote the direction of magnetization and electric polarization along *c*, which was revealed by macroscopic magnetization and polarization measurements. The fractions of the C_A and C_B domains are always comparable to those of the Q_A and Q_B domains.

Supplementary Note 1 | The transformation properties of the order parameters.

Supplementary Eqs. 1–7 given below were developed by considering how the magnetization distribution transforms under the symmetry operations given in Supplementary Table 1, similar to the procedure used in Supplementary Ref. 1.

$$m_x P_z = P_z,\ m_y P_z = P_z,\ m_z P_z = -P_z, \quad (1)$$

$$m_x X_1 = X_1,\ m_y X_1 = X_1,\ m_z X_1 = X_1, \quad (2)$$

$$m_x X_3 = -X_3,\ m_y X_3 = -X_3,\ m_z X_3 = X_3, \quad (3)$$

$$m_x M_{\mathrm{QA}}^{\mathrm{D1}} = e^{i(k_x+k_y)/2} M_{\mathrm{QB}}^{\mathrm{D1}^*},\ m_y M_{\mathrm{QA}}^{\mathrm{D1}} = e^{ik_y/2} M_{\mathrm{QB}}^{\mathrm{D1}},\ m_z M_{\mathrm{QA}}^{\mathrm{D1}} = e^{ik_x/2} M_{\mathrm{QA}}^{\mathrm{D1}}, \quad (4)$$

$$m_x M_{\mathrm{QA}}^{\mathrm{D2}} = e^{i(k_x+k_y)/2} M_{\mathrm{QB}}^{\mathrm{D2}^*},\ m_y M_{\mathrm{QA}}^{\mathrm{D2}} = -e^{ik_y/2} M_{\mathrm{QB}}^{\mathrm{D2}},\ m_z M_{\mathrm{QA}}^{\mathrm{D2}} = -e^{ik_x/2} M_{\mathrm{QA}}^{\mathrm{D2}}, \quad (5)$$

$$m_x M_{\mathrm{QB}}^{\mathrm{D1}} = e^{i(k_x-k_y)/2} M_{\mathrm{QA}}^{\mathrm{D1}^*},\ m_y M_{\mathrm{QB}}^{\mathrm{D1}} = e^{-ik_y/2} M_{\mathrm{QA}}^{\mathrm{D1}},\ m_z M_{\mathrm{QB}}^{\mathrm{D1}} = e^{ik_x/2} M_{\mathrm{QB}}^{\mathrm{D1}}, \quad (6)$$

$$m_x M_{\mathrm{QB}}^{\mathrm{D2}} = e^{i(k_x-k_y)/2} M_{\mathrm{QA}}^{\mathrm{D2}^*},\ m_y M_{\mathrm{QB}}^{\mathrm{D2}} = -e^{-ik_y/2} M_{\mathrm{QA}}^{\mathrm{D2}},\ m_z M_{\mathrm{QB}}^{\mathrm{D2}} = -e^{ik_x/2} M_{\mathrm{QB}}^{\mathrm{D2}}. \quad (7)$$

The mirror (or glide) operations, m_x, m_y, and m_z, are the minimum symmetry elements for the point group *Pnma*, because all the other operations ($\bar{1}$, 2_x, 2_y, and 2_z) can be generated by the product of these mirror (or glide) operations. Note that the transformations of axial vectors such as the magnetization, magnetic order parameters, or the angular momentum $\mathbf{r} \times \mathbf{p}$ are different from those of polar vectors such as position **r** and momentum **p**. For axial vectors, the result for transformations involving a change in handedness (e.g. mirror or inversion operation) includes an extra factor of (−1).

Supplementary References

1. Harris, A. B., Kenzelmann, M., Aharony, A. & Entin-Wohlman, O. Effect of inversion symmetry on the incommensurate order in multiferroic $R\mathrm{Mn_2O_5}$ (R = rare earth). *Phys. Rev. B* **78**, 014407 (2008).
